# Study on the influence of hole defects with different shapes on the mechanical behavior and damage law of coal and rock

**DOI:** 10.1371/journal.pone.0265753

**Published:** 2022-03-22

**Authors:** Tao Wang, Qinrong Kang, Xiangyang Zhang, Xiangxi Xu, Wenpu Li, Huan Zhang

**Affiliations:** 1 School of Safety and Emergency Management Engineering, Taiyuan University of Technology, Taiyuan, Shanxi, China; 2 Key Laboratory of Safety and High-efficiency Coal Mining, Ministry of Education (Anhui University of Science and Technology) Huainan, China; 3 Engineering Research Center of Phosphorus Resources Development and Utilization of Ministry of Education, Wuhan Institute of Technology, Wuhan, China; 4 Shandong Jinling Mining Limited By Share Ltd, Zibo, China; NUST: National University of Sciences and Technology, PAKISTAN

## Abstract

To study the effect of different shapes of hole defects in coal and rocks on their mechanical behavior and macro damage law, the microscopic mechanical parameters required for particle flow code (PFC) simulation were calibrated with laboratory test data, and then the evolution process of crack and stress field in coal and rocks with circle, square, triangular and trapezoidal holes under uniaxial compression were researched. The findings indicate that: the existence of hole defects lowers the elastic modulus, peak stress, peak strain and other mechanical parameters of coal and rock, and the reduction degree is influenced by the shape of defect. Meanwhile, the existence of hole defects promotes the generation and evolution of meso-cracks in coal and rock. For coal and rock with hole defects, the crack initiation stress and expansion stress are less than those of intact coal and rocks. The crack initiation stress and expansion stress of coal and rocks with trapezoidal hole defects are the smallest, and the coal and rocks with circular hole defects are the largest. The existence of hole defects weakens the damage degree of coal and rocks to some extent. With the increase of axial strain, the evolution curve of the number of meso-cracks shows stage characteristics, which consists of the calm period before the crack initiation point, the stable growth stage between the crack initiation point and the dilatation point, and the accelerated growth stage after the dilatation point. Before the initiation of crack, the concentration zone of compressive stress is located on the left and right sides of the hole defect, and the concentration zone of tensile stress is located on the upper and lower sides of the hole defect. The concentration of tensile stress is the main reason for the initiation and propagation of cracks, while the existence of compressive stress chain among macroscopic cracks is the cause of the residual strength of coal and rocks after failure.

## Introduction

Affected by geological process or engineering disturbance, natural coal and rocks contains various defective structures such as cracks and holes [1, 2]. The instability failure of rock engineering goes hand in hand with the expansion and evolution of internal defects [3, 4]. Therefore, making a profound study of damage evolution, deformation and mechanical properties of rock with defects is significant for ensuring the safety and stability of all kinds of rock engineering.

Scholars from home and abroad have did lots of research on the mechanical properties and fracture law of defective rocks, mainly centering on the mechanical behavior and damage law of defective rocks with prefabricated cracks and prefabricated circular holes. In the study on the mechanical damage law of rocks with prefabricated cracks, Gratchev et al. promoted uniaxial compression tests on three types of rock samples with cracks of different sizes, and it was concluded that the length and width of rock joints were important parameters affecting the strength of rock mass [5]. Liu et al. made prefabricated cracks in the roof mudstone, studied the effect of prefabricated crack angle and size on the mechanical properties and failure mode of coal-rock combination samples, and concluded that the parameters of prefabricated cracks in the roof rock have an important influence on the effect of roof weakening [6]. Cao et al. researched the crack propagation and evolution law of rock with two and three prefabricated crack defects with the help of indoor tests, and determined seven crack penetration modes according to the different inclination of rock bridge [7]. Li et al. studied the effect on inclination angle of the prefabricated crack on mechanical properties and acoustic emission parameters of coal samples under uniaxial compression, and believed that the existence of cracks reduced the mechanical parameters of coal and rock, and the change of acoustic emission fractal dimension could be used as a warning indicator for the occurrence dynamic disasters of coal and rock [8]. Wang et al. promoted quasi-static loading tests on SCB coal samples with prefabricated cracks, and found that the fracture mode and fracture toughness of the samples were affected by both bedding and prefabricated crack direction. The fracture toughness decreased with the decrease of the angle between the bedding and loading direction, and the inclined prefabricated crack further weakened the ability of the samples to resist crack propagation [9]. With the help of discrete element PFC software, Yang et al. studied the mechanical properties, crack initiation, propagation and penetration behavior of rocks with non parallel double fractures and three fractures. They believe that the mechanical parameters and crack evolution law of rocks are greatly affected by the crack defect inclination angle. The rock initiation stress and damage stress increase first and then decrease with the increase of crack inclination angle [10, 11]. Lee et al. did research on the crack initiation and evolution law of rock with crack defects by using PFC software, and designed the geometry of the fracture defect by the combination of a horizontal defect and an inclined defect below it [12]. Huang et al. did research on the mechanical behaviors and crack penetration modes of three kinds of prefabricated fractured sandstone samples under triaxial compression by laboratory test and numerical method [13].

In the research of mechanical damage law of coal and rocks with circular hole defects, by using acoustic emission technology and moment tensor method, Liu et al. studied the development process of crack of the granite specimen with central hole under uniaxial compression [14]. Meng et al. carried out uniaxial compression tests on large-size samples with double holes, and analyzed the effect of hole spacing on the mechanical parameters and acoustic emission characteristics of the samples, along with the crack initiation and propagation evolution law of the samples with double holes [15]. Huang et al. did study on the strength failure behavior and crack evolution mechanism of granite with three non coplanar circular holes, analyzed four typical crack coalescence modes, and revealed the crack evolution mechanism around the existing holes in granite [16]. Zhang et al. promoted uniaxial compression tests of briquette coal with hole, analyzed the distribution characteristics of strain localization zone around round hole at different loading stages by means of digital speckle method, and explored the relationship between strain localization zone characteristics and macroscopic cracks [17]. Lai also conducted the uniaxial compression test of brittle coal samples with holes, and obtained that the crack development and failure form of coal samples with holes are more complex. As initial defect, the holes degrade the strength of coal samples, change the duration of energy storage and release of coal samples, reduce the accumulation and release rate of elastic properties, and reduce the severity of peak failure [18]. By using RFPA 2D software, Tang et al. did simulation of the crack initiation and propagation process of rock with circular holes under static and dynamic loads [19, 20]. Wang et al. conducted a research on the change law of the impact liability of drilled coal samples, and believed that drilling could reduce the strain energy accumulated before the peak of coal samples, reduce the energy released per unit time after the peak of coal samples, and slow down the post-peak energy release and energy dissipation rate of samples [21]. By means of CT scanning, Zhang et al. did a study on the influence of the number and aperture of holes on the spatial evolution of cracks in marble during compression, and proposed that in the process of specimen failure, with the increase of the diameter or number of holes, the tensile crack and shear crack increase, while the far field crack decrease [22]. Wang et al. did a uniaxial compression simulation test of rock samples with prefabricated cracks and holes by PFC2D, and analyzed the effects of prefabricated cracks and holes on crack initiation and propagation evolution from a mesoscopic perspective [23].

Fruitful research results have been achieved in the study on the mechanical properties and damage law of rock with defects from the above analysis, mainly focusing on the influence of physical parameters such as the number and relative position of cracks or circular holes on the mechanical properties of rock, while there are few reports on the effect of the shape of holes on the mechanical behavior of coal and the macro-micro fracture mechanism. Therefore, based on the test results of raw coal samples by uniaxial compression, this paper first calibrates the meso parameter values required for PFC simulation, and then provides a detailed analysis of the influence of hole defects with different shapes on the mechanical parameters of coal and rocks, the initiation and propagation process of meso cracks and the macro failure modes of coal and rocks.

## Model establishment and parameter calibration

### Contact model

The failure of coal and rock mass is the result of crack initiation, propagation and evolution of internal microstructure under the action of external stress. The bonded-particle model (BPM) is proposed by Potyondy et al., which is specially used to simulate the meso damage process of rock materials in the particle flow code (PFC) [24, 25]. In PFC, there are two kinds of particle bonding models, namely contact bond model (CBM) and parallel bond model (PBM). The CBM can be assumed as an elastic spring (or punctate cement) with constant normal and shear stiffness acting on the contact point of two particles, which is similar to the point cement material connecting two adjacent particles, the point contact determines that it can only resist tensile and shear effects, but not rotation between particles, and the particle contact stiffness still plays a role after bond failure. The parallel bonding model like the physical behavior of cement-like substances connecting two adjacent particles, and its effect is similar to a beam resisting the moment caused by particle rotation or shear in the bonding area. The stiffness of parallel bonding model is affected by bonding stiffness and contact stiffness. After bonding failure, the bonding stiffness fails, and the contact stiffness still works. Therefore, the parallel bond model is more suitable to simulate the damage and failure process of rock materials. By using the parallel bond model, the mechanical behavior and damage law of coal and rock with hole defects of different shapes is simulated in this paper.

### Model establishment

**Fig 1:**
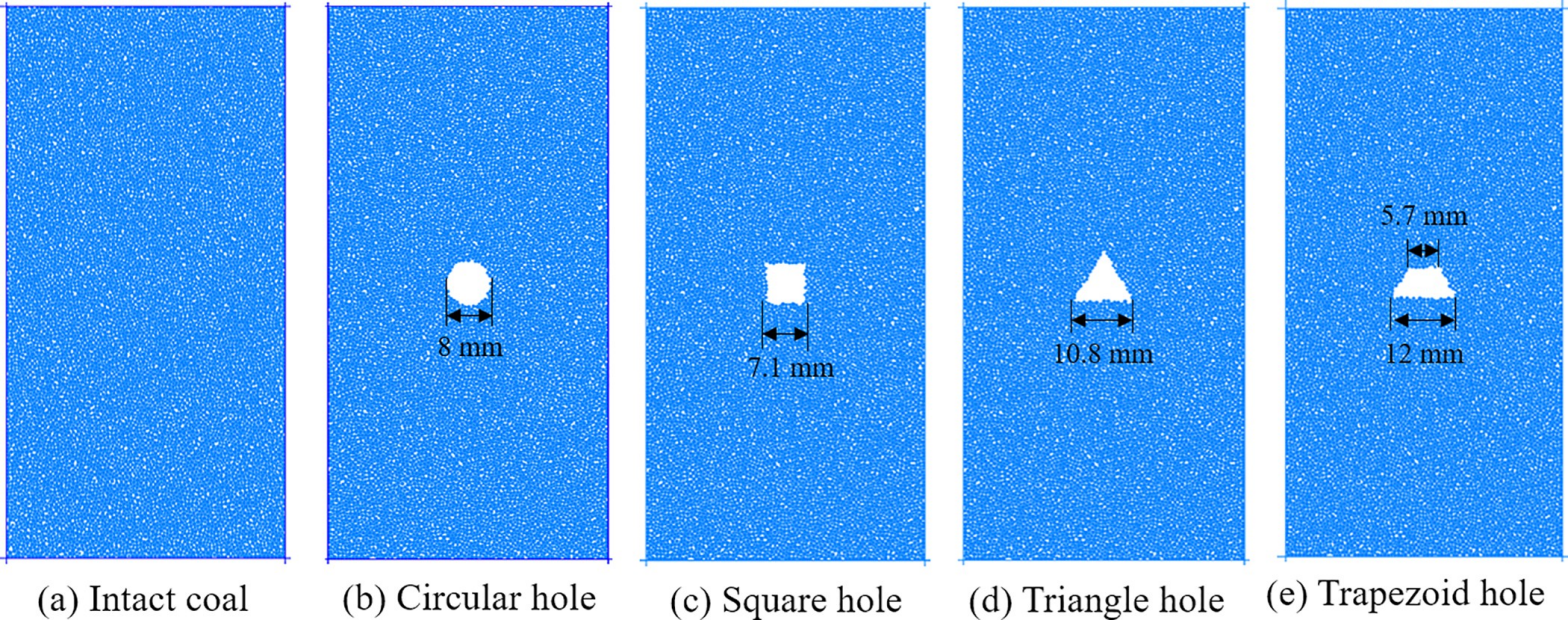
Numerical calculation model.

To study the effect of hole defect shapes on mechanical properties and damage law of coal and rock, five numerical calculation models are established, including complete coal and rock samples, including circular, square, triangular and trapezoidal hole defect samples. The width of coal and rock sample is 50mm and the height is 100mm. The boundary excavation method is used to prepare the hole defects with different shapes. Firstly, the boundary line of hole defects is drawn in AutoCAD, then the boundary line is imported into PFC software, and the hole defects can be formed by deleting the particles inside the boundary line. For avoiding the influence of hole size on the simulation results, the defect areas of holes with different shapes are approximately equal, of which the circular area is 50.24 mm^2^, the square area is 50.41 mm^2^, the triangle area is 50.22 mm^2^, and the trapezoid area is 50.45 mm^2^. Fig 1 shows the established numerical calculation model. The model loading is realized by the opposite movement of the upper and lower walls. The moving speed of the upper and lower walls is set to 0.02m/s. Reading the existing research results for reference, the moving speed of the wall meets the requirements of static loading [26, 27]. The axial stress, axial strain and transverse strain of coal samples during loading can be monitored by setting measuring circle.

### Determination of meso-parameters

**Fig 2:**
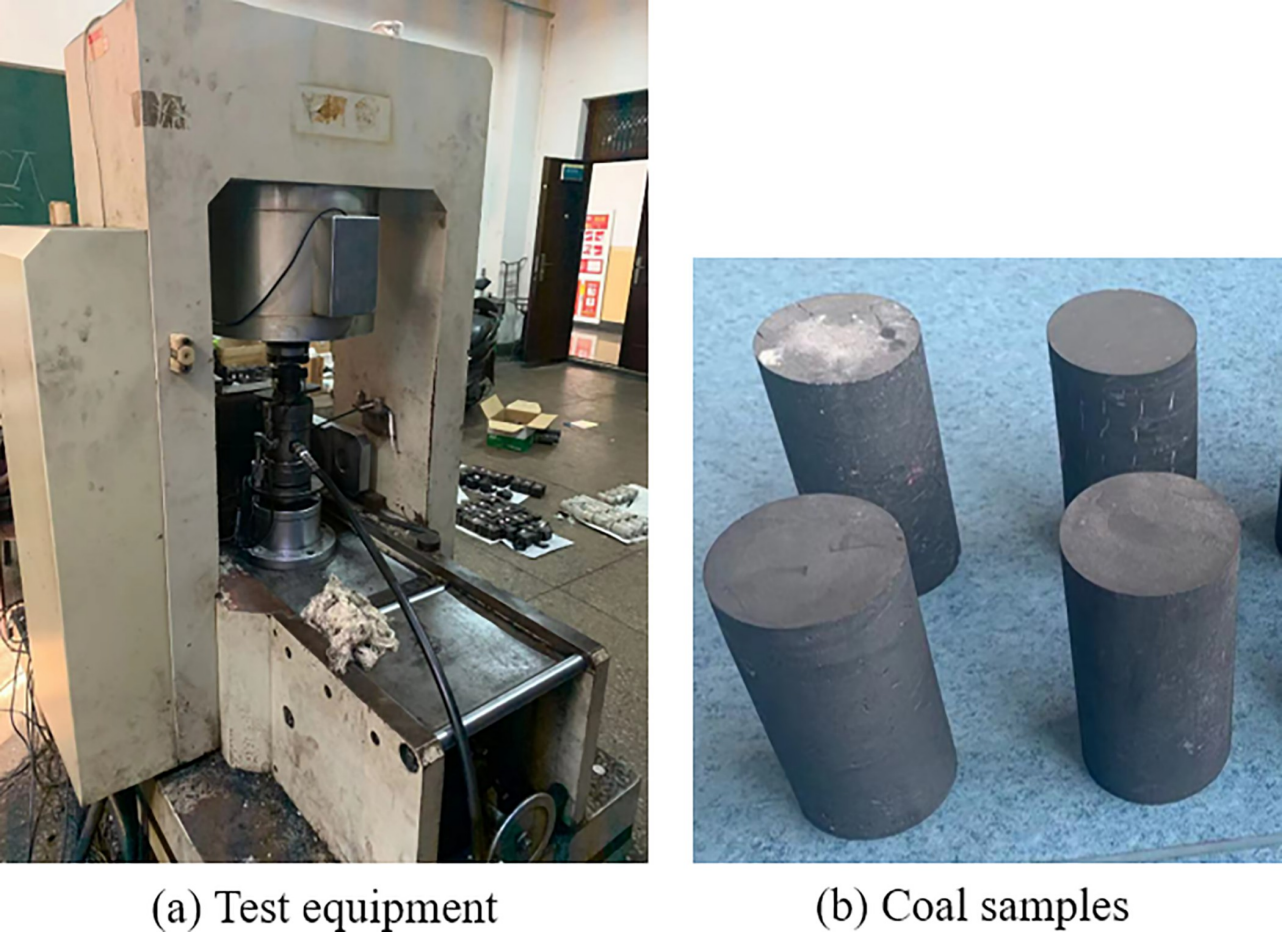
Test equipment and coal samples.

**Fig 3:**
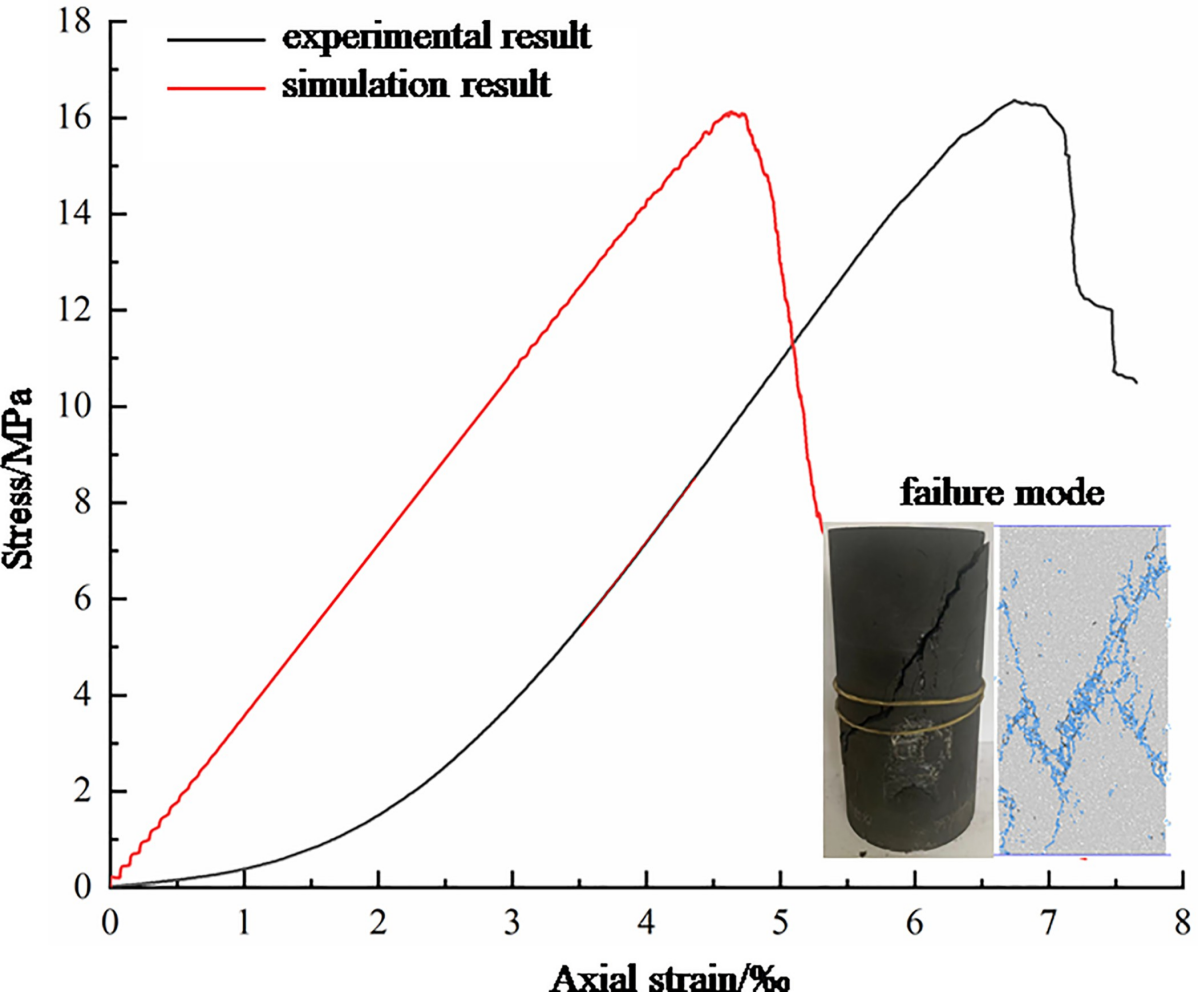
Comparison of stress-strain curves and failure modes of indoor test and numerical simulation.

In the parallel bonding model, the micro parameters such as contact modulus, stiffness ratio and bonding strength affect the macro mechanical behavior and damage fracture results of coal and rock. The determination of meso-parameters is the prerequisite for obtaining accurate model results with the help of discrete element method. The calibration of meso-parameters often adopts the "trial and error method", that is, the value of simulation meso-parameters continuously adjusted until the obtained simulation macro-mechanical parameters match the results of the indoor test. Therefore, this article first carried out a uniaxial compression test of a complete raw coal sample. Large blocks of coal for preparing coal samples are taken from Hequ open pit coal mine, which is sited in Xinzhou City, Shanxi Province, China. The 8~14# coal seams are minable, the production scale is 10 million t/a, and the ore field area is 24.9536 km^2^. Standard coal samples are prepared by coring and grinding. The sample specification is 50mm in diameter and 100mm in height (Fig 2B). Using the RMT-150B electro-hydraulic servo as testing equipment (Fig 2A) and adopting the displacement loading mode, the loading speed is 0.1mm/min. In the test, the lateral displacement of coal sample is monitored by displacement meter, and the axial displacement of coal sample is recorded by pressure machine. On the basis of the definition of strain, the transverse strain and axial strain of coal sample are obtained, and the Poisson’s ratio of coal sample is obtained after that. To avoid the influence of primary pores and cracks on laboratory test results, ultrasonic testing is carried out on the primary coal samples to ensure that the ultrasonic velocity difference between the coal samples does not exceed 100m/s. Four coal samples are used in the indoor testing, and the Poisson’s ratio, peak stress and elastic modulus of each coal sample have little difference. The mechanical parameters of one coal sample are taken as an example for parameter calibration. The stress-strain curves and macroscopic failure modes of coal and rocks are gained through the test, as shown in Fig 3.

By adjusting the values of meso parameters continuously, a group of meso parameters are obtained, which makes the numerical simulation results consistent with the indoor test results. Table 1 shows the comparison of mechanical parameters of the numerical simulation result and the laboratory test result, and Fig 3 shows the comparison of the stress-strain curves and the failure modes. From Fig 3 and Table 1, we will see that the elastic modulus, peak stress and Poisson’s ratio of coal samples obtained in laboratory test are not much different from those obtained in numerical simulation, and the macroscopic crack distribution patterns of coal samples obtained in laboratory test and numerical simulation are basically the same. From Fig 3 we can also see that there is a large gap between peak strain of coal samples obtained from laboratory test and numerical simulation. The raw coal sample has abundant joints and fissures, and the initial loading stage of the laboratory test is primary pore and fracture closure stage. During the numerical simulation, the sample is pre-compressed and the pores are basically compacted. There is no initial compaction stage or the displacement value in the initial compaction stage is very small. The existence of the initial compaction stage is the main reason for the large difference between the peak strain obtained from laboratory testing and numerical simulation. In conclusion, the calibrated meso-mechanical parameters can be used to solve the numerical simulation. The values of meso parameters used in the numerical model are shown in Table 2. The uniaxial compression tests of coal and rock with holes and defects of different shapes are carried out by using the values of meso parameters in Table 2.

**Table 1:** Mechanical parameters of intact specimens obtained from laboratory test and numerical modeling.

Mechanical parameters of intact coal	Uniaxial compressive strength (MPa)	Elastic modulus (GPa)	poisson ratio
Experimental result	16.36	3.62	0.21
Simulation result	16.12	3.57	0.203
Deviation	1.47%	1.36%	3.3%

**Table 2:** Meso-mechanical parameters used in PFC2D simulation.

Micro-parameters	Values
Minimum particle radius *R*_min_/m	2.5×10^−4^
Maximum to minimum particle radius ratio	1.66
Particle density*ρ*/(kg/m^3^)	2700
Particle friction coefficient*μ*	0.1
Young’s modulus of the particle *E*_*c*_/GPa	4.1
Ratio of normal to shear stiffness of the particle kn/ks	1.4
Parallel bond coefficient radius*λ*	1
Young’s modulus of the parallel bond *E*_*c*_/GPa	4.1
Ratio of normal to shear stiffness of the parallel bond ${\bar{k}}_{n}/{\bar{k}}_{s}$	1.4
porosity	0.14
Parallel bond tensile strength ${\bar{\sigma }}_{c}/\mathrm{MPa}$	17
Parallel bond cohesion $\bar{c}/\mathrm{MPa}$	17
Parallel bond internal friction angle $\bar{\varphi }/({}^{\circ})$	30

## Analysis of test results

### Influence of defect types on mechanical parameters of coal and rock

**Fig 4:**
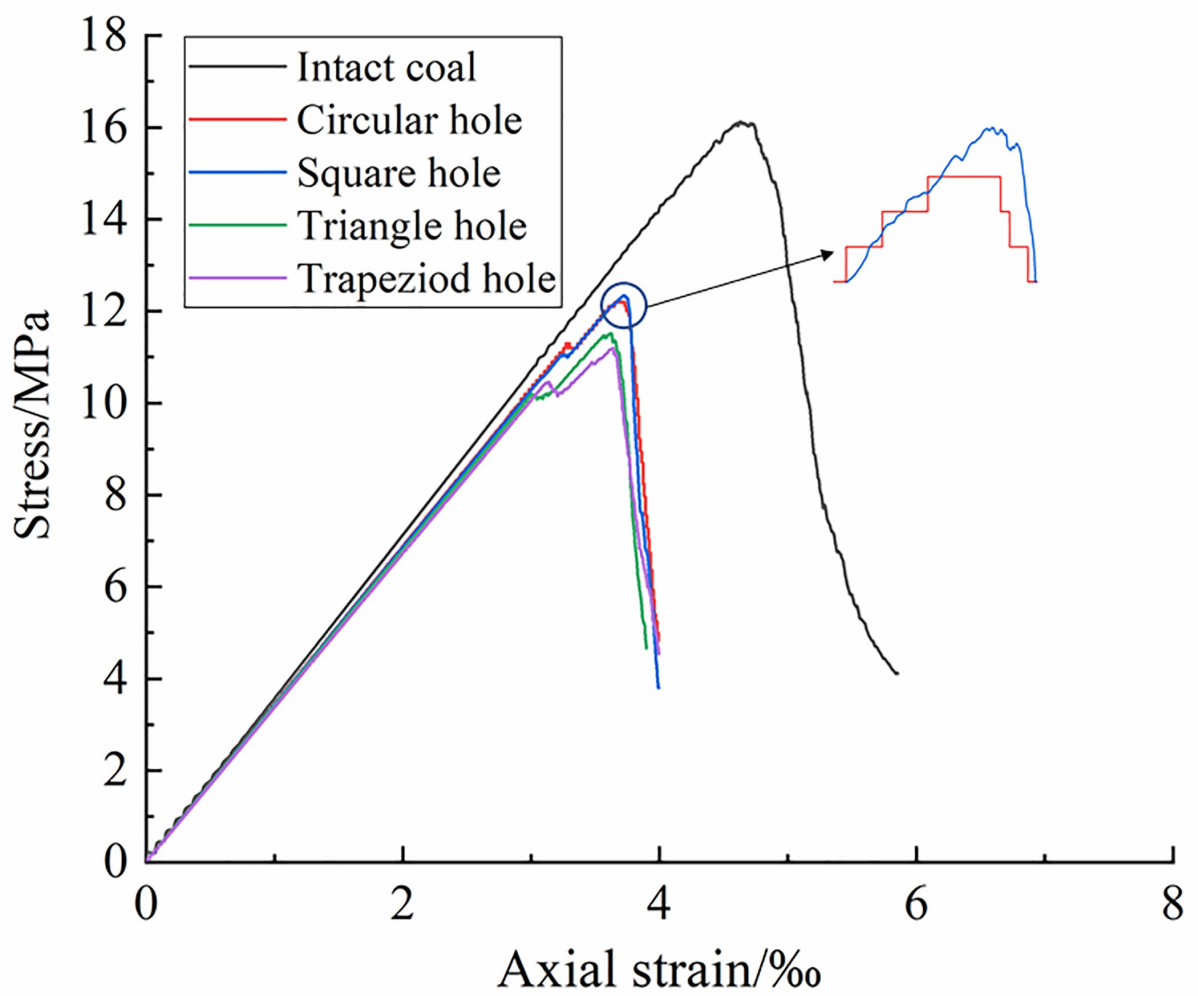
Stress-strain curves of coal and rocks with hole defects of different shapes.

Fig 4 shows the stress-strain curves of coal and rocks with hole defects of different shapes. The trend of stress-strain curve of intact coal and rock is basically the same as that of coal and rocks with hole defects of different shapes. The stress-strain curves of coal and rocks with hole defects of different shapes have obvious stress fluctuation in the plastic yield stage before the peak stress, while for complete coal and rocks, it’s relatively smooth. The peak stresses of coal and rocks with hole defects of different shapes are obviously less than those of intact coal and rocks.

The uniaxial compression mechanical parameters of coal and rocks with hole defects of different shapes are shown in Table 3, the existence of defects in coal and rocks will have a certain implication on its mechanical parameters, and different defect shapes have different effects on mechanical parameters of coal and rocks.

**Table 3:** Mechanical parameters of coal and rocks with varying shaped holes defects.

Mechanical parameters	Peak stress /MPa	Peak strain	Elastic modulus /GPa
Intact coal	16.12	0.00464	3.56
Circular hole	12.20	0.00365	3.44
Square hole	12.34	0.00372	3.43
Triangle hole	11.52	0.00362	3.41
Trapezoid hole	11.20	0.00364	3.37

**Fig 5:**
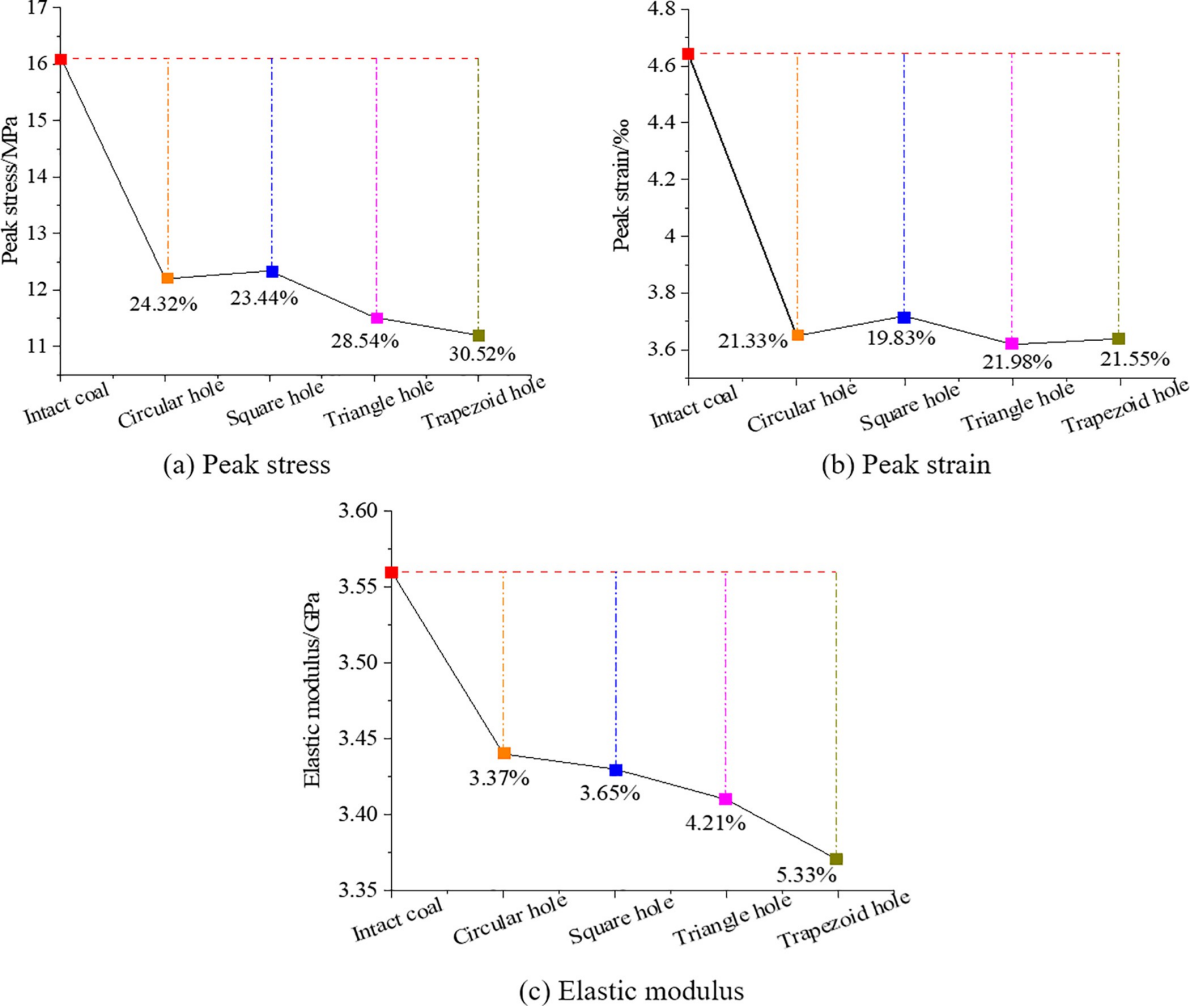
Mechanical parameters of coal and rocks with holes defects of different shapes.

Fig 5 shows the variation laws of elastic modulus,peak stress and peak strain of coal and rocks with different hole defects. In Fig 5(A), we can see that compared with the complete coal and rock, the peak stress of coal and rock with trapezoidal hole defects decreases the most, about 30.52%. The peak stress of coal and rocks with square hole defect decreases the least, about 23.44%. Compared with intact coal and rocks, the peak stresses of defective coal and rocks with circular hole and triangular hole are reduced by 24.32% and 28.54% respectively. The maximum difference of peak stress loss of intact coal and rock caused by different hole shape defects is about 23.17%.

In Fig 5(B), we can see that compared with the complete coal and rocks, the peak strain of coal and rock with triangular hole defects decreases the most, about 21.98%. The decrease of peak strain of coal and rock with square hole defect is the smallest, about 19.83%. The decrease of peak strains of coal and rocks with circular and trapezoidal hole defects are 21.33% and 21.55% respectively. As we can see, the hole defects of different shapes have an impact on the peak strains of intact coal and rocks, but there is little difference among them, and the maximum is only 9.8%.

In Fig 5(C), we can see that compared with the intact coal and rock, the elastic modulus of coal and rocks with trapezoidal hole defects decrease the most, about 5.33%. The decrease of peak strain of coal and rock with circular hole defect is the smallest, about 3.37%. The decreases of peak strain of coal and rocks with square and triangular holes are 3.65% and 4.21% respectively. As we can see, the hole defects of different shapes have an impact on the peak strain of intact coal and rocks, but the influence values vary greatly, the maximum is about 36.8%.

Through the above analysis, it will be found that the existence of hole defects will affect the peak stress, peak strain and elastic modulus of coal and rocks to some extent, and the influence range is related to the shape of hole defects, among them, trapezoidal hole has the greatest impact on the peak stress and elastic modulus of coal and rock, and triangular hole has the greatest impact on the peak strain of coal and rock.

### Effect of defect type on crack initiation stress and damage stress

**Fig 6:**
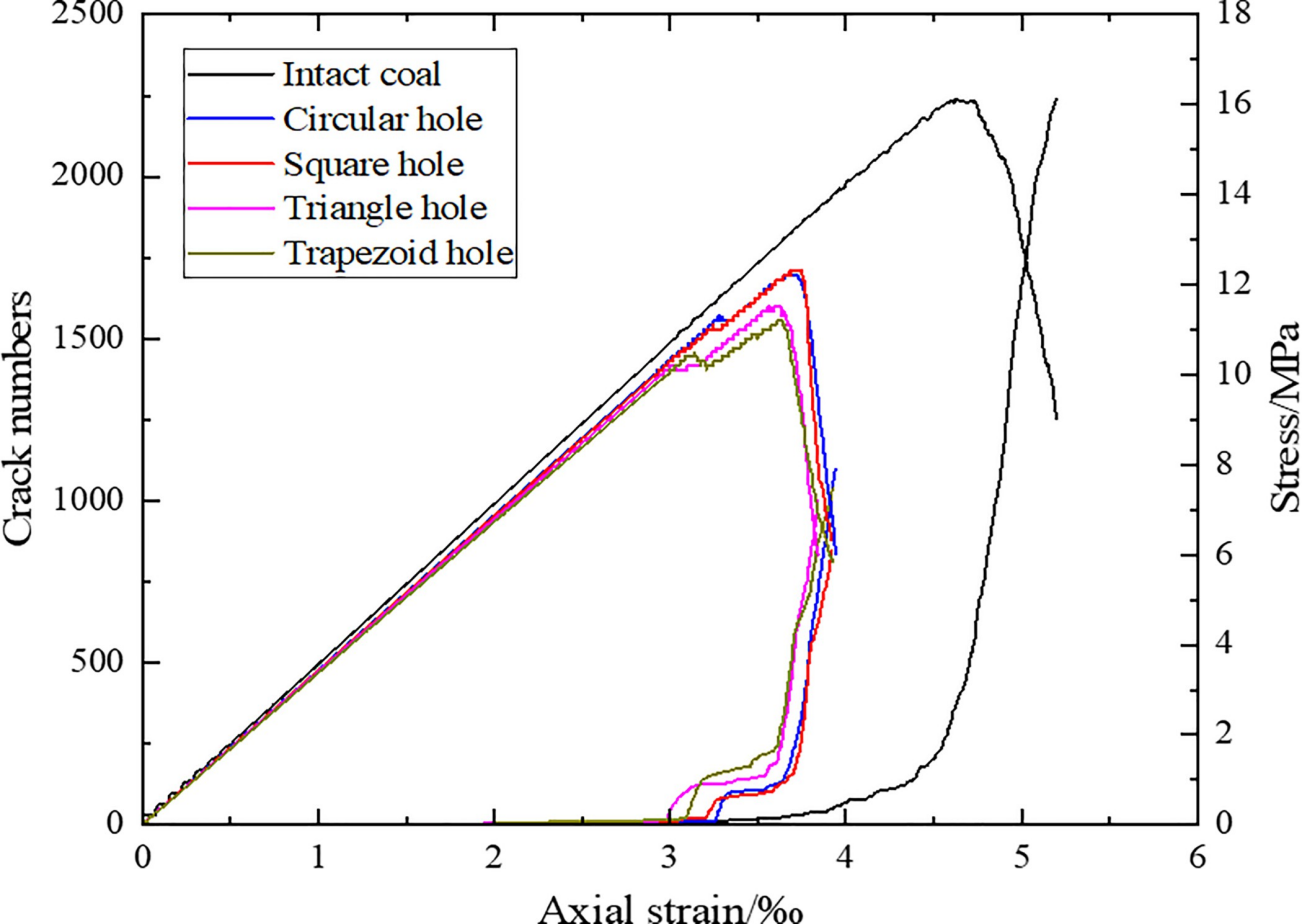
Relationship between stress, crack number and axial strain of coal and rocks with hole defects of different shapes.

Fig 6 shows the stress-strain curves and the numbers of strain-crack curves of coal and rocks with hole defects of different shape and intact coal and rock. In Fig 6,we can see that the number of coal and rock cracks gradually increases with the increase of axial strain, which consists of three stages, namely, the quiet period before the crack initiation point, the stable propagation stage from the crack initiation point to the expansion point, and the accelerated propagation stage after the expansion point. After the capacity expansion point, the number of micro cracks increases significantly, and micro cracks rapidly produce, expand and penetrate at this stage, and the macro cracks is produced.

The evolution curve shapes of the number of cracks in coal and rocks with holes of different shapes are basically the same, there is a stepped transition between the initiation point and the expansion point, however, the number of cracks of evolution curve of intact rock is relatively smooth. This may be due to the existence of hole defects that cause stress concentration around the defect. When the stress value exceeds the micro-tensile strength of the particle, the multiple micro-cracks will quickly occur around the defect, and the slope of the crack growth curve will increase rapidly. With the further increase of the axial stress, the cracks propagate to the inside of the specimen, and the number of cracks of the evolution curve is consistent with the evolution law of a complete rock.

**Fig 7:**
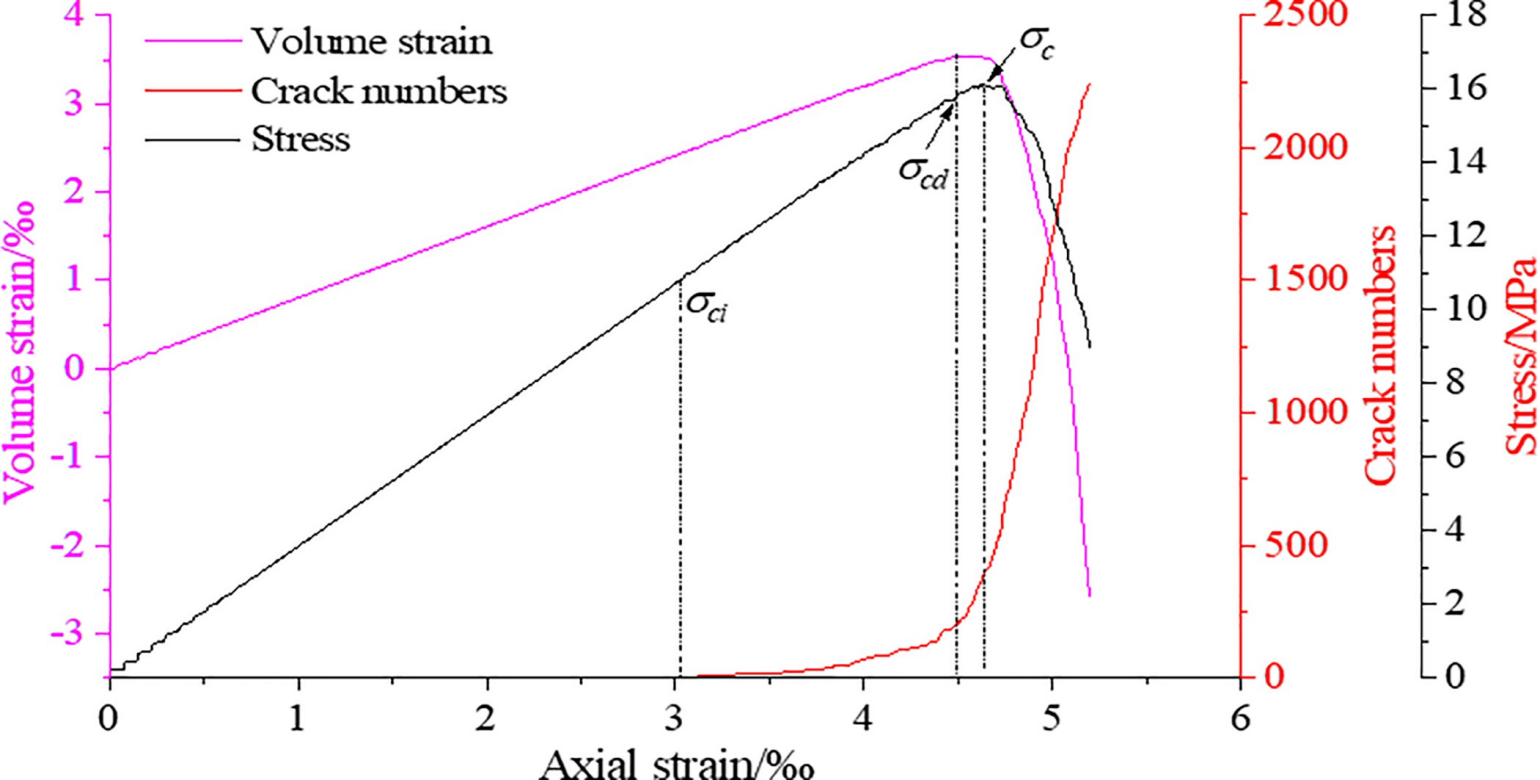
Calibration of crack initiation stress and dilatation stress of coal and rocks.

Potyondy gave a method to determine the rock initiation stress based on the number of microscopic cracks: first determine the number of cracks *m*_*c*_ at the peak stress of the sample, and then find the stress corresponding to the number of cracks of 1% *m*_*c*_ as the simulated crack initiation stress value *σ*_*ci*_. In the volumetric strain curve, the appearance of the inverted point marks the expansion of the rock volume, which is the result of the intersection of the fractures. The stress corresponding to the inverted point of the volumetric strain curve is the dilatation stress. Taking a complete coal and rock as an example, with the aid of the above method, the calibration of the coal and rock initiation stress and dilatation stress during the loading process is shown in Fig 7. Table 4 shows the crack initiation stress value, the dilatation stress value, the strain values corresponding to the crack initiation stress and the dilatation stress, the relationship between the crack initiation stress and the peak stress, and the relationship between the dilatation stress and the peak stress of coal and rocks with hole defects of different shapes.

**Table 4:** Crack initiation stress and dilatation stress values of coal and rocks.

Defect shape	Crack initiation stress/*σ*_*ci*_	Crack initiation strain /*ε*_*ci*_	Volume dilatation stress /*σ*_*cd*_	Volume dilatation strain /*ε*_*cd*_	*σ*_*ci*_ */σ*_*c*_	*σ*_*cd*_ */σ*_*c*_
Intact coal	10.81	0.00303	15.76	0.00449	67.1%	97.8%
Circular hole	8.41	0.00244	11.20	0.003269	68.9%	91.8%
Square hole	7.95	0.00232	11.00	0.003217	64.4%	89.1%
Triangle hole	6.37	0.00187	10.40	0.00299	55.3%	90.3%
Trapezoid hole	5.62	0.00167	10.10	0.003104	50.2%	90.2%

**Fig 8:**
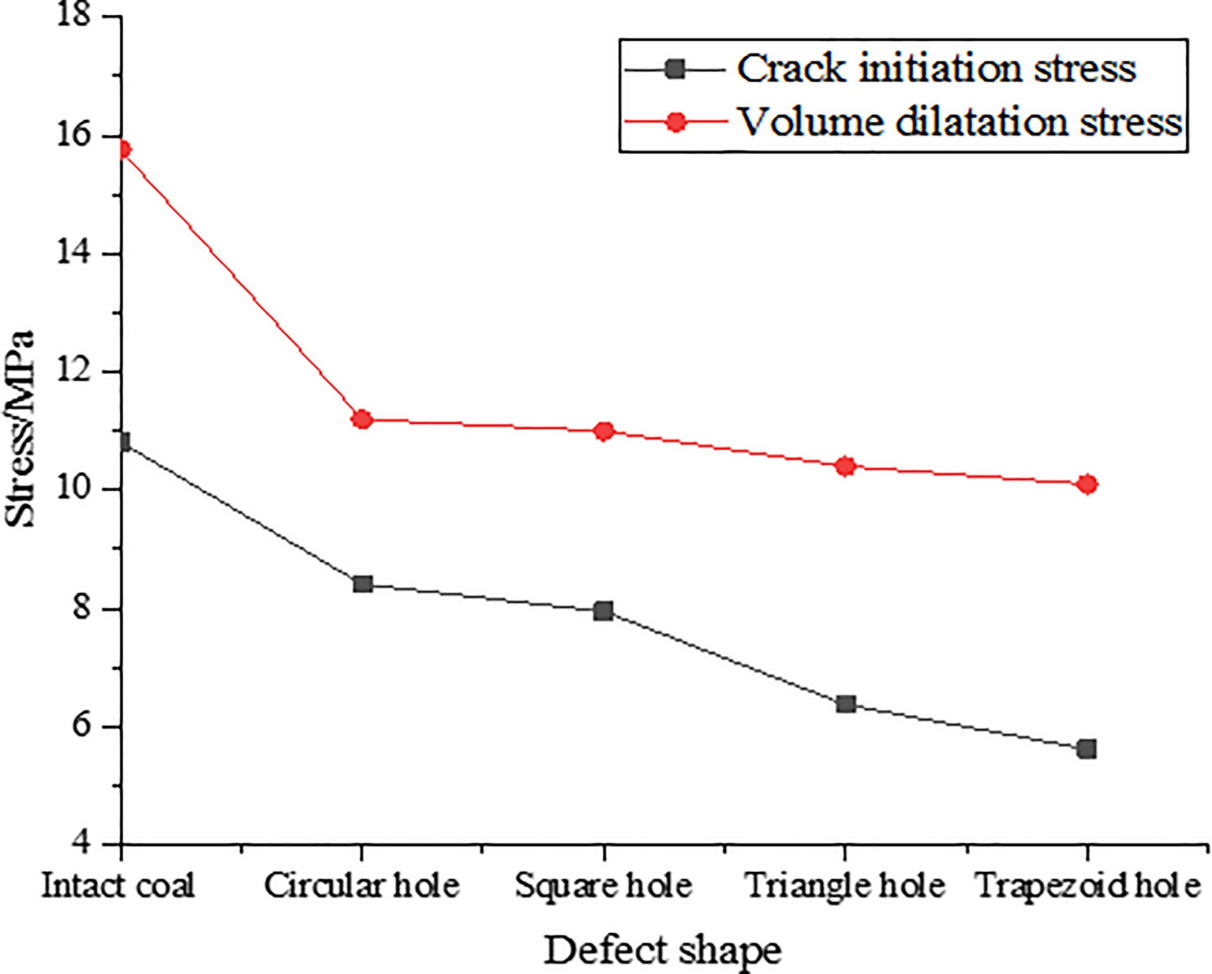
Crack initiation stress and dilatation stress of coal and rocks with holes defects of different shapes.

According to the data in Table 4, the relationship curves between initiation stress and dilatation stress of complete coal and coal with different shapes of pores are drawn, as shown in Fig 8.

As we can see, Table 4 and Fig 8 show that the initiation stress and dilatation stress of coal and rocks with different hole defects are smaller than those of complete coal and rocks, among them, the initiation stress and dilatation stress of coal and rocks with trapezoidal hole defects are the smallest, and coal and rocks with circular hole defects are the largest. It shows that when the coal and rock contains sharp-angled defects, microcracks are more likely to form inside the coal rock mass.

### Effect of defect shape on macroscopic failure mode of coal and rock

**Fig 9:**
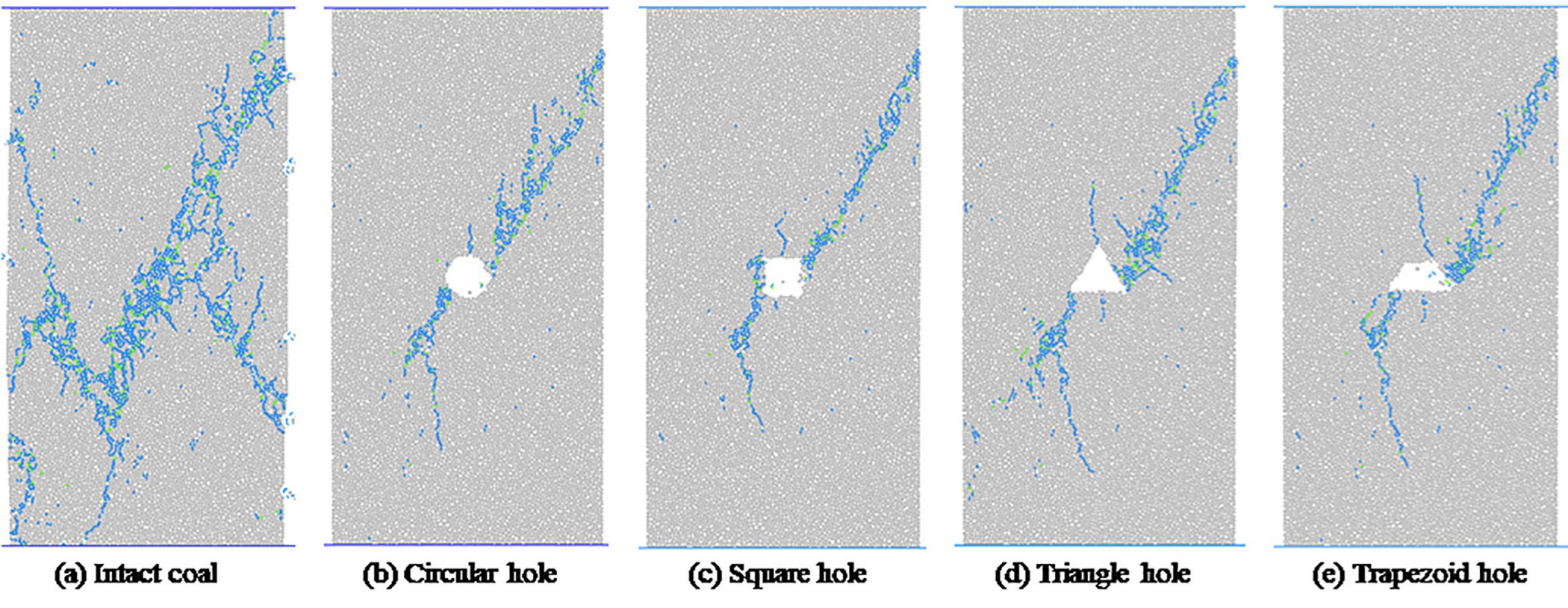
Simulation results of macroscopic failure modes of coal and rocks with hole defects of different shapes.

Fig 9 shows the macroscopic failure mode of intact coal and coal with pore defects of different shapes at 80% *σ*_*c*_ after the peak. The blue line represents the meso-tensile failure, and the green line represents the meso-shear failure. In Fig 9, we can see that under uniaxial compression, intact coal and coal with different shapes of pores mainly undergo macro-shear failure, and macroscopic shear cracks are mainly caused by microscopic tensile failure, which is the macroscopic shear failure caused by microscopic tensile fracture. In addition to the formation of a main oblique cut crack after the failure of the complete coal and rock under uniaxial compression, a secondary crack evolves on each side of the main crack. Through uniaxial compression, only one main control crack is formed after the coal with hole defect is destroyed, and the shape of the main control crack in coal with hole defects of different shapes is similar. The macroscopic fracture morphology of intact coal is obviously different from that of coal with pore defects, and the shapes of pore defects have little effect on the macroscopic fracture morphology of coal under uniaxial compression.

**Fig 10:**
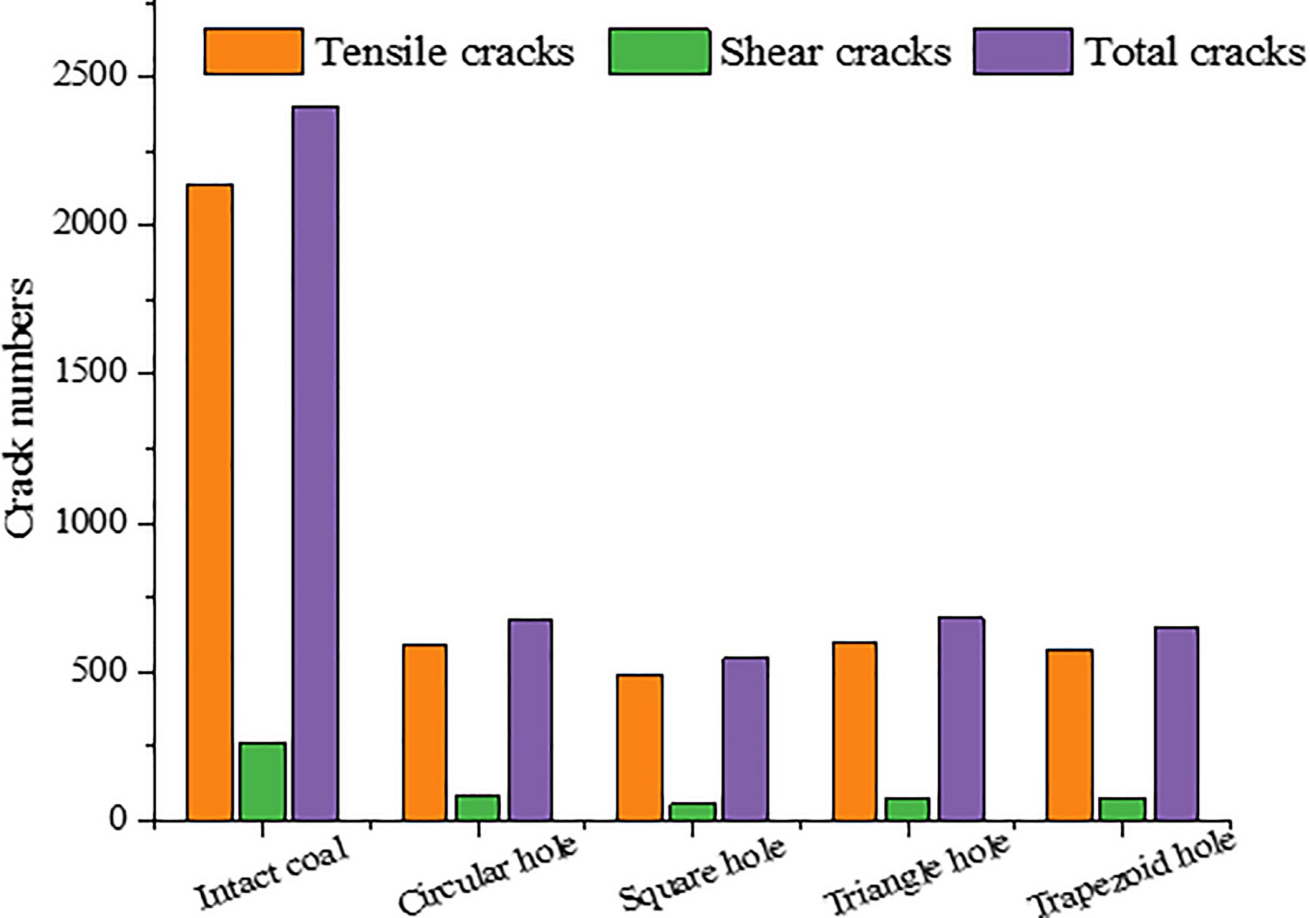
Number of micro-cracks in coal and rocks with hole defects of different shapes.

The number of microscopic cracks can reflect the extent of coal rock failure [28, 29]. Although coal and rocks with pore defects of different shapes have roughly the same crack shapes when they are macroscopically destroyed, the damage degrees are not the same. The number of microscopic cracks in the model at a stress level of 80% *σ*_*c*_ after the peak is shown in Fig 10. As we can see, the numbers of meso-tensile cracks and shear cracks in intact coal and rock are much larger than those in coal and rocks with pore defects, indicating that the damage degree of intact coal and rock under uniaxial compression is higher than that of coal and rock with holes and defects. The number of microscopic cracks in coal and rocks with pore defects of different shapes are also different, indicating that the shape of pore defects has a certain influence on the damage degree of coal and rocks. Among them, the total number of cracks in coal and rock with triangular hole is the highest, about 28.4% of the complete coal and rock, while the total number of cracks in coal and rock with square hole is the least, about 22.8% of the complete coal and rock.

## Influence of defect types on damage evolution law of coal and rock

**Fig 11:**
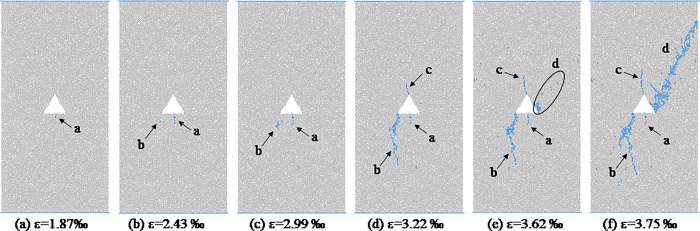
Meso-damage process of coal and rock with triangular hole defect.

**Fig 12:**
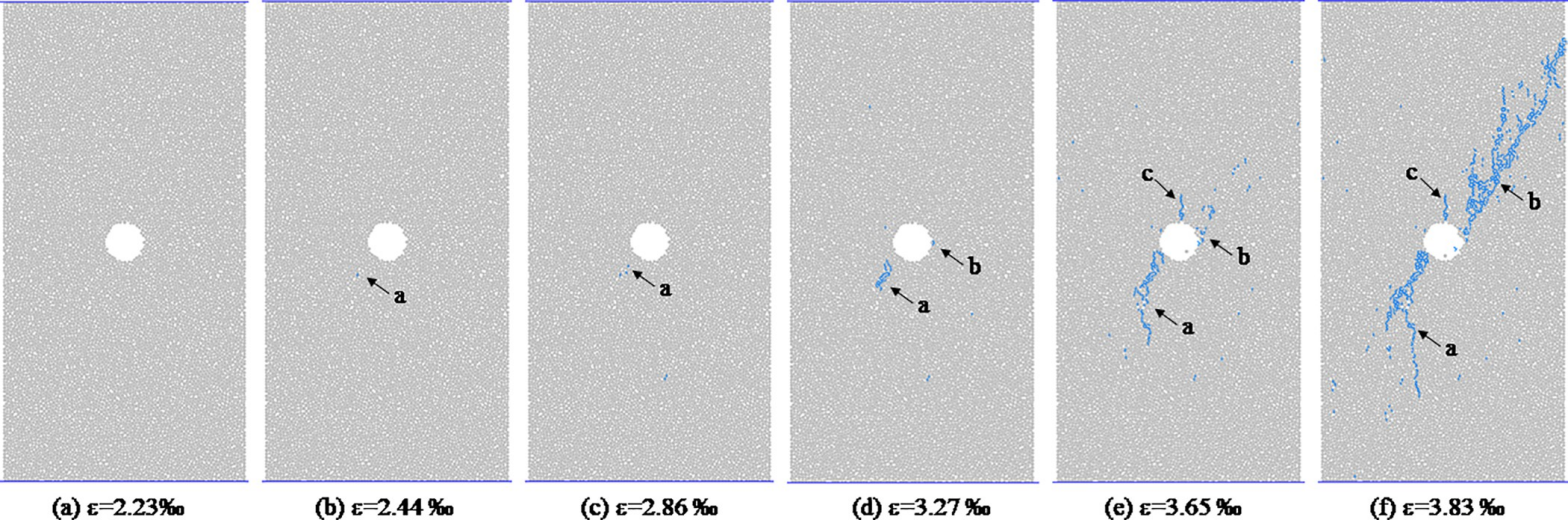
Meso-damage process of coal and rock with circle hole defect.

**Fig 13:**
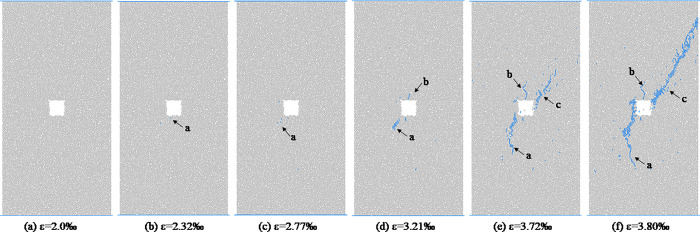
Meso-damage process of coal and rock with square hole defect.

**Fig 14:**
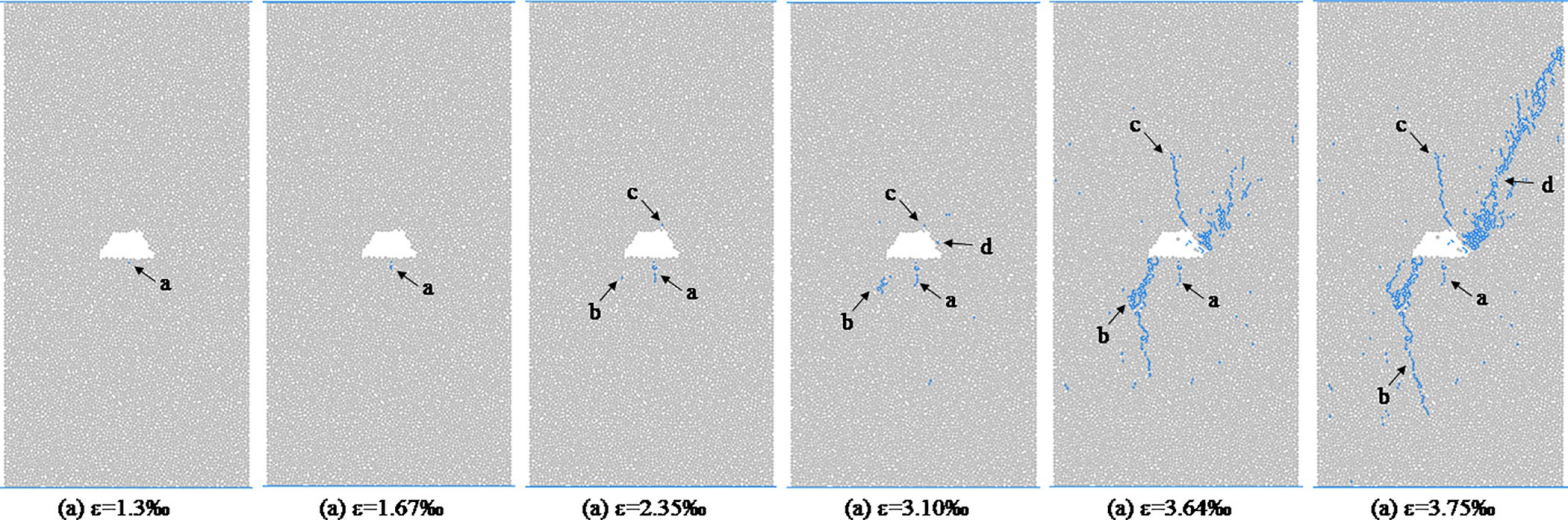
Meso-damage process of coal and rock with trapezoid hole defect.

From the analysis in the previous section, we will found that the shapes of hole defects have little effect on the macro failure modes of coal and rocks. In this section, the coal and rocks with triangle hole defects are used as an example to analyze the propagation process of micro-crack form initiation to macro-fracture. Fig 11 shows the evolution process of meso-cracks in coal and rock with triangle pore defects at different loading moments. As we can be see from Fig 11, when the stress value reaches the crack initiation stress, the sample first cracks around the triangular hole defect, forming a meso-tensile crack a, which is located in the middle of the bottom edge of the triangular defect. With the increase of axial stress, the crack a gradually expands downward, and the expansion direction is roughly parallel to the loading direction. When the axial strain reaches 0.00234, a crack b is formed near the sharp corner at the lower left of the triangular defect. At the dilatation stress point, the specimen mainly undergoes the propagation and evolution of cracks a and b. At the moment before the peak, crack a no longer changes, crack b continues to expand downward, and the direction is deflected during the expansion process, from the initial inclined angle expansion direction to the vertical expansion direction roughly parallel to the loading direction. During the downward propagation of crack b, crack c is formed at the top corner of the triangular defect, and the pixel propagation direction of crack c is roughly parallel to the loading direction. At the peak moment, cracks a, b, and c stop expanding in the vertical direction, and the scattered distribution of meso cracks is formed in area d, and the cracks are densely distributed in the area close to the triangular defect. After the peak point, bifurcation cracks are formed at the turning point of crack b, and the cracks in area d penetrate each other to form macroscopic cracks, and the specimen is destroyed. Figs 12–14 respectively show the initiation and propagation process of microcracks in the coal and rocks with circular, square and trapezoidal defects. From Figs 11–14, we can see that although the macro failure modes of coal and rocks with defects of different shapes are the same, the micro crack evolution processes are slightly different. There is a certain distance between the initial microcrack initiation position of coal with circular defect, and the crack gradually expands to the circular defect position after the crack initiation. The initial cracks in coal with triangular, square and trapezoidal defects all start from the periphery of the defects, and then spread away from the defects.

**Fig 15:**
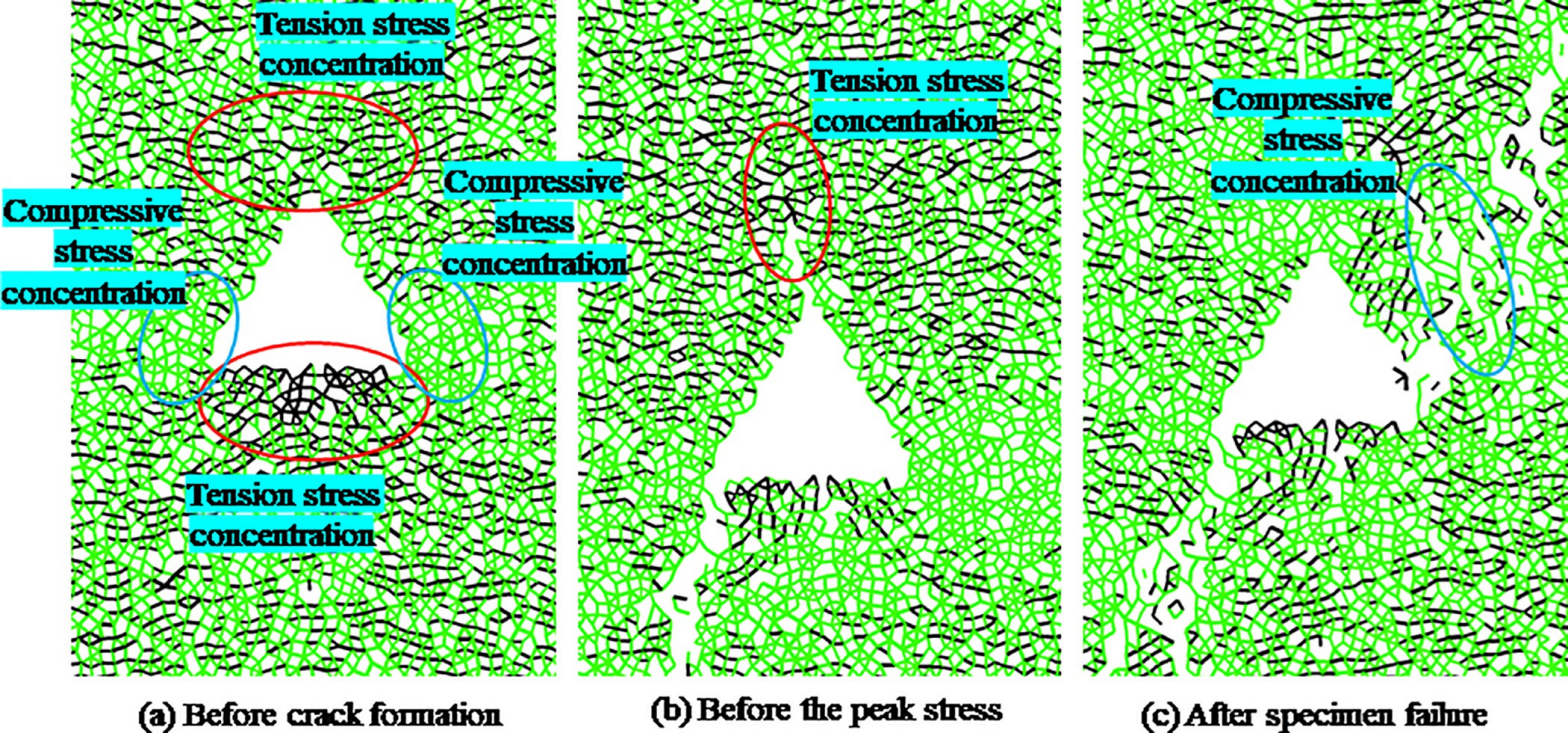
Stress chain diagram of rock model with triangular hole defect before and after crack generation.

In order to analyze the evolution process of meso crack initiation and propagation, Fig 15 shows the stress chain diagram of coal and rock with triangular hole defects before and after failure, the green line represents compressive stress and the black line represents tensile stress. From Fig 15A, we can see that before crack initiation, there is an obvious stress concentration around the triangular defect, and there is also an obvious tensile stress concentration near the bottom edge and top angle of the triangle. The tensile stress concentration at the bottom is greater, while there is obvious compressive stress concentration on both sides of the triangle. With the increasing axial stress, the tensile stress of the defect at the bottom of the triangle first exceeds the tensile strength between the particles, and the crack starts from this area first (Fig 11A). In Fig 15B, we can see that after crack initiation, there is an obvious tensile stress concentration at the tip of crack, and the tensile stress direction is roughly parallel to the horizontal direction, leading to the crack propagation direction roughly parallel to the loading direction (crack c in Fig 11D). In Fig 15C, we can see that there is an obvious compressive stress concentration chain among the macro cracks after the failure of the sample, and the existence of the compressive stress chain is the main reason for the residual strength of coal and rock after failure.

## Conclusions

1. The existence of defects in coal and rocks is important related factor to the mechanical parameters, such as elastic modulus, peak stress, peak strain. Meanwhile, the influence levels of different defects shapes on the mechanical parameters of coal and rocks are different.
2. The numbers of meso cracks in coal and rocks gradually increase with the increase of axial strain, which consists of three stages: the quiet period before the crack initiation point, the stable propagation stage from the crack initiation point to the expansion point, and the accelerated propagation stage after the expansion point. In the coal and rocks with hole defects of different shapes, the numbers of cracks of evolution curves have a stepped transition between the initiation points and the expansion points of cracks. However, in the complete coal and rock, the number of cracks of evolution curve is relatively smooth.
3. The initiation stress and dilatation stress of coal and rocks with different hole defects are smaller than those of complete coal and rocks, among them, the initiation stress and dilatation stress of coal and rock with trapezoidal hole defect are the smallest, and the coal and rock with circular hole defect are the largest.
4. Under uniaxial compression, the damage degree of intact coal and rock is higher than that of coal and rocks with hole defects. The shapes of hole defects have a certain effect on the damage degree of coal and rocks. Tensile stress concentration is the main reason for crack initiation and propagation, and the existence of compressive stress chain between macro cracks is the cause of residual strength after coal and rock failure.
